# Guide to Enhancing Swallowing Initiation: Insights from Findings in Healthy Subjects and Dysphagic Patients

**DOI:** 10.1007/s40141-018-0192-y

**Published:** 2018-05-23

**Authors:** Kensuke Yamamura, Masayuki Kurose, Keiichiro Okamoto

**Affiliations:** Division of Oral Physiology, Department of Oral Biological Sciences, Niigata University Graduate School of Medical and Dental Sciences, 2-5274 Gakkocho-dori, Chuo-ku, Niigata, 951-8514 Japan

**Keywords:** Swallowing, Swallowing initiation, Stimulation, Plasticity, Dysphagia, Review

## Abstract

**Purpose of Review:**

Difficulty in initiating swallowing is one of the main symptoms of oropharyngeal dysphagia. Therefore, enhancing swallowing initiation is an important approach for the treatment of oropharyngeal dysphagia. This review aims to introduce recent approaches to enhancing swallowing and to discuss their therapeutic potential.

**Recent Findings:**

Both central interventions such as non-invasive brain stimulation and peripheral interventions such as electrical stimulation to peripheral tissues are conducted to enhance swallowing. Recent studies have paid more attention to generating neuroplasticity to produce long-lasting facilitative effect on swallowing.

**Summary:**

Transcranial magnetic stimulation (TMS), transcranial direct current stimulation (tDCS), pharyngeal electrical stimulation (PES), transcutaneous electrical stimulation, and somatic and chemical stimulation were introduced. Considerable evidence supports the therapeutic potential of TMS and PES. Other approaches need further studies to verify their efficacy (e.g., duration of the effect and a limit of effectiveness) and/or possible risk of adverse effects.

## Introduction

Swallowing is the first stage of digestion when food or liquid is transported from the mouth to the stomach and consists of two stages; the oropharyngeal and esophageal swallowing [1, 2]. The oropharyngeal swallowing also includes a function protecting the respiratory tract from the swallowed materials. Oropharyngeal dysphagia frequently leads to pulmonary aspiration resulting in aspiration pneumonia. It also leads to reduced food and fluid intake resulting in malnutrition and dehydration and seriously affects systemic health and reduces quality of life. Difficulty in initiating swallowing is one of the main symptoms of oropharyngeal dysphagia. Therefore, establishment of a technique enhancing swallowing initiation is an important approach for the treatment of oropharyngeal dysphagia.

The oropharyngeal swallowing is reflexively induced and regulated by the brainstem neural networks, the so-called swallowing central pattern generator (swallowing CPG). The swallowing CPG is further divided into the dorsal swallowing group (DSG) including a part of the nucleus tractus solitarius (NTS) and the ventral swallowing group (VSG) including the reticular formation around the nucleus ambiguus. Activation of DSG triggers the swallowing reflex, and the information is sent to VSG, which sends efferent fibers to the motor nuclei of the Vth, VIIth, IXth, Xth, and XIIth cranial nerves innervating more than 25 muscles involved in swallowing [3, 4]. It is now accepted that DSG receives peripheral sensory inputs from oral, pharyngeal, and laryngeal mucosa as well as from higher brain regions such as the cerebral cortex, and both inputs play a crucial role in initiating and modulating swallowing. Therefore, theoretical approaches enhancing oropharyngeal swallowing include enhancement of central and/or peripheral facilitative inputs to the swallowing CPG. This review aims to introduce recent approaches enhancing swallowing in healthy subjects and dysphagic patients, and their therapeutic potential is discussed.

## Central Interventions Increasing Facilitative Inputs

Classical electrophysiological studies demonstrated that electrical stimulation of the cerebral cortex in anesthetized subprimates [5, 6] and awake primates [7] induces swallowing. Progress in brain imaging techniques enabled demonstration that many cortical and subcortical areas such as the primary sensorimotor cortex, premotor, supplementary motor areas and anterior cingulate, frontoparietal operculum, and the insula cortex are activated in relation to swallowing [8–10]. In addition, inactivation or lesion of the cerebral cortex disrupts swallowing in awake primates and patients [11–13]. Furthermore, activation of the cerebral cortex using transcranial magnetic stimulation (TMS) in man induces swallowing as well as contraction of the upper esophageal sphincter [14]. These findings suggest that the cerebral cortex is a theoretical target to enhance facilitative inputs to the swallowing CPG. One of the attractive innovations in developing interventions for dysphagia in this decade is enhancing central inputs over long periods using non-invasive brain stimulation techniques such as TMS and transcranial direct current stimulation (tDCS).

## Transcranial Magnetic Stimulation (TMS)

TMS is a technique activating axons in the cerebral cortex, cerebellum, and subcortical white matter by small eddy currents generated by a magnetic field through an intact skull. The magnetic field can be generated with a large (more than 5 kA) but very brief (less than 1 ms) electrical current passing through a figure of-eight coil, which is set directly above the subject’s head [15•]. To give recognition to the therapeutic potential of TMS for dysphagia, Hamdy and colleagues as well as other researches have spent more than two decades. During this period, they have established a sophisticated experimental setup utilizing three different kinds of TMS.

One is single-pulse TMS. Single-pulse TMS to the cortical motor area evokes a transient muscle contraction, which can be recorded by electromyogram (EMG), and such EMG activity was termed the motor evoked potential (MEP). Since amplitude of MEPs reflects functional facilitative connectivity between the motor cortex and the motoneurons in the brainstem (i.e., corticobulbar excitability), single-pulse TMS is used for cortical mapping to define functional representations of the cortical motor area. Single-pulse TMS is also used as a test stimulus, to quantitatively evaluate corticobulbar excitability by comparing amplitude of MEPs between conditions. In early studies, Hamdy and colleagues conducted cortical mapping using single-pulse TMS to find out topographic representation of swallowing musculatures in healthy subjects and dysphagia patients, and found somatotopical representation of mylohyoid, pharyngeal, and oesophageal musculature on the motor and premotor areas of both hemispheres [16]. They noticed the asymmetrical representation of such areas between the hemispheres, which was independent of handedness. The finding had led the idea that swallowing dominant and non-dominant hemispheres exist. The idea was also supported by the study in dysphagic and non-dysphagic patients after unilateral hemispheric stroke, showing that TMS stimulation of the unaffected hemisphere evoked smaller pharyngeal responses in dysphagic patients than in non-dysphagic patients, indicating the weaker corticobulbar excitability (i.e., cortex to swallow-related region in the medulla) in the unaffected hemisphere of the dysphagic patients. This in turn suggests that dysphagia occurs when the dominant hemisphere is damaged [17]. They also found an expansion of cortical representation of the pharyngeal musculature in the undamaged hemisphere when the patients were recovering from post-stroke dysphagia [16, 18]. They then developed a concept of plasticity of the neural networks regulating swallowing during the recovery, and their research focus converged to develop interventions facilitating a neural plasticity which may occur during natural recovery process from dysphagia after unilateral brain damage.

Another type of TMS is low-frequency (e.g., 1 Hz for 10 min) but high intensity (e.g., 120% of the threshold of MEPs when the subject is at rest) repetitive transcranial magnetic stimulation (rTMS). Such low-frequency TMS inhibits corticobulbar excitability up to 45 min [19], and this technique was termed “virtual lesion.” Virtual lesion could be considered as an establishment of dysphagia model after unilateral brain damage to verify the efficacy of various interventions using healthy subject before they are tested in dysphagia patients.

The other type of TMS is high-frequency (e.g., 5 Hz, 250 pulses) rTMS. High-frequency rTMS to the motor cortex in the unaffected (i.e., not virtual lesioned) hemisphere reversed the effect of the virtual lesion and the effects lasted for up to 50 min [20]. Thereafter, high-frequency rTMS was considered excitatory rTMS as a possible intervention to enhance swallowing [15•]. After that, many clinical studies have been conducted to test the effects of rTMS on swallowing performance and/or severity of dysphagia in post-stroke dysphagic patients. There are disparities in the intervention protocols such as targets of rTMS (e.g., affected or unaffected hemisphere), lesion type, time from diagnosis between studies by different groups, and further studies will be needed for a definitive conclusion of the efficacy of rTMS. Nonetheless, significant results were achieved by using rTMS in most studies. ([15, 21, 22•, 23] for recent reviews).

Recently, rTMS with various frequencies (1, 5, 10, and 20 Hz) were applied to the cerebellar cortex to the pharyngeal motor area in 17 healthy subjects [24]. Results showed that effect was frequency specific, and high-frequency rTMS (10 Hz, 250 pulses) increased cortico-pharyngeal MEP amplitudes (i.e., pharyngeal corticobulbar excitability) up to 30 min and suggested therapeutic potential of cerebellar rTMS for dysphagia.

## Transcranial Direct Current Stimulation (tDCS)

tDCS is a technique modulating the resting membrane potential of neurons by a weak direct electric current (1 to 2 mA) using a pair of electrodes, which is put directly over the scalp. Studies have shown that anodal tDCS stimulation depolarizes and thereby increases excitability of neurons, whereas cathodal stimulation hyperpolarises the neuron and reduces its excitability. Nitsche et al. [25–27] suggested that cortical stimulation by tDCS induces neuroplastic changes by altering NMDA (N-methyl-D-aspartate) receptor activity. As a noninvasive brain stimulating device, tDCS has advantages for its portability, ease of use, low costs, and targeting deeper regions of the brain compared to TMS. Therefore, many clinical studies in dysphagia patients as well as basic studies in healthy subjects have been done in this decade.

Application of tDCS to test the effect on swallowing was firstly conducted by Hamdy and colleagues in 17 healthy subjects [28]. After mapping the cortex using single-pulse TMS, they stimulated the pharyngeal motor area by tDCS in various stimulation protocols, and found that anodal tDCS at 1.5 mA for 10 min and that at 1 mA for 20 min increased cortical excitability in the stimulated hemisphere up to 60 min and that cathodal tDCS decreased cortical excitability. They then studied the effects of anodal tDCS (20 min, 1 mA) to the pharyngeal motor area on cortical activity defined by magnetoencephalography and on swallowing performance during three different (simple, fast, and challenged) volitional swallowing tasks in 21 healthy subjects. They found a significant bilateral enhancement of cortical swallow-related activation during fast swallowing following left tDCS and during challenged swallowing following right tDCS. They also found slight improvements in swallowing performance during challenged swallowing (increase in the number of correctly timed swallows within a predetermined time window) following left tDCS [28]. Although the effect of tDCS on swallowing performance was small in healthy subjects, it became clearer when “virtual lesion” was applied in 15 healthy subjects [29]. They showed that anodal tDCS (1.5 mA, 10 min) applied contralateral to the “virtual lesioned” hemisphere increased pharyngeal MEPs bilaterally reversing the inhibitory effects of virtual lesion and prominent enhancement in swallowing behaviors particularly in swallow precision up to 60 min. The finding indicates therapeutic potential for dysphasic patients following unilateral brain damage (e.g., stroke).

To date, several clinical studies have been conducted in post-stroke dysphagic patients to test the effects of anodal dTCS. Kumar et al. studied the effects of anodal tDCS (2 mA for 30 min/day, 5 days) to the unaffected hemisphere in 14 acute phase stroke patients with dysphagia, and showed that tDCS induced better improvement in Dysphagia Outcome and Severity Scale (DOSS) than sham stimulation [30]. Yang et al. [31] and Shigematsu et al. [32] studied the effects of anodal dTCS to the affected hemisphere (1 mA for 20 min, a total of ten tDCS sessions for 2 weeks) together with conventional swallowing therapy in post-stroke dysphagic patients (*n* = 16 for Yang et al. and *n* = 20 for Shigematu et al.). Results showed that tDCS induced better improvements in functional dysphagia scale (FDS) at 3 months after tDCS [31] or in DOSS lasting for 1 month [32]. Effect of bihemispheric anodal tDCS (1 mA for 20 min, a total of ten tDCS sessions within 2 weeks) was tested by Ahn et al. [33] in 26 post-stroke dysphagic patients and reported significant improvement in the patients who received tDCS but not in the sham group. These findings suggest that stimulating either hemisphere by anodal tDCS improve dysphagia and utility of this technique as an effective intervention for dysphagia rehabilitation. However, further clinical studies are needed to verify the efficacy of tDCS and the risk of adverse effects; basic studies to clarify properties of induced neural changes in the affected and unaffected hemispheres are also expected.

## Inhibitory Effects on Swallowing Induced by Brain Stimulation

Several studies were conducted in this decade showing that electrical stimulation of localized brain region inhibits swallowing. In experimental animals, electrical stimulation of the red nucleus [34] and anterior part of the cortical masticatory area (A-area) [35] inhibited the swallowing reflex induced by electrical stimulation of the superior laryngeal nerve (SLN) in anesthetized rats. Tsujimura et al. also showed that stimulation of A-area inhibited cortically-evoked swallows by stimulation of cortical swallowing area within the insular cortex, and the inhibitory effect was significantly more than that on SLN-evoked swallows [36]. In man, Sörös et al. showed that stimulation of the right inferior posterior insular cortex at 4 mA induced irregular and delayed swallows with a perception of a “stutter in swallowing” in a 24-year-old patient with medically intractable stereotyped nocturnal hypermotor seizures due to a ganglioglioma [37]. These findings reconfirmed that inhibitory inputs from higher brain to the swallowing CPG exist.

## Peripheral Interventions Enhancing Swallowing Initiation

Sensory inputs related to swallowing are generated by tactile, thermal and chemical stimuli, and travel via sensory fibers in the trigeminal (Vth), the facial (VIIth), the glossopharyngeal (IXth), the internal branch of the superior laryngeal (iSLN), and other branches of the vagus (Xth) nerves. Particularly, stimulation of receptive fields innervated by iSLN or IXth such as the epiglottis and arytenoids, the lateral pharyngeal wall, posterior tonsillar pillar, and peritonsillar areas [38] are reported to be effective in triggering or modulating the swallowing reflex. Not only triggering swallowing, sensory inputs are also conveyed by ascending pathways to higher regions in the brainstem, subcortical, and cortical levels (see [39] for review), and suggested to induce sustained alternation (i.e., neuroplasticity) in the central nervous system (e.g., [40]). Recent studies have paid more attention to the potential importance of sensory inputs in generating neuroplasticity within the central nervous system regulating swallowing (see [41] for review).

## Pharyngeal Electrical Stimulation (PES)

Hamdy and colleagues studied the effects of pharyngeal electrical stimulation (PES) using bipolar-ring intraluminal electrodes (10 Hz train of stimuli for 10 min) on corticobulbar excitability evaluated by pharyngeal MEPs evoked by single-pulse TMS [42] and showed that PES induced sustained increase in MEPs for 30 min. The finding suggested that PES induced neuroplasticity in the swallowing motor cortex. They then tried to find an optimal stimulation parameter inducing a facilitative neuroplasticity in corticobulbar excitability in eight healthy subjects and found that largest facilitative effect was induced by 5 Hz PES at 75% maximum tolerated intensity (20 ± 4 mA, 0.2-ms duration for each pulse), which was correlated with an increase in the size of swallow-related area in the cortex. On the contrary, stimulation of higher than 10 Hz decreased excitability [43]. In the same study, they also found that 5 Hz PES at 75% maximum tolerated intensity (16 ± 4 mA) increased corticobulbar excitability mainly in the undamaged hemisphere together with an improvement in swallowing function (reduced pharyngeal transit times, swallowing response times, and aspiration scores) in 16 post-stroke dysphagic patients [43]. Furthermore, they showed that 5 Hz PES induced similar effects in “virtual lesion” model in 13 healthy subjects: 5 Hz PES (9.7 ± 0.7 mA) reversed the inhibitory effects on MEPs induced by virtual lesion, and improved swallowing performance during challenged swallowing (increase in the number of correctly timed swallows within a predetermined time window) [44]. In the same study, they also applied 5 Hz PES to 50 post-stroke dysphagic patients and showed that PES (14.4 ± 1.3 mA) conducted one or three times a day improved swallowing function (airway protection, aspiration, and feeding status). They then concluded that one stimulation session per day for 3 days would be effective intervention [44]. More recently, they studied the effects of 5 Hz PES (10 min at 75% of maximum tolerated intensity) on cortical activity during volitional swallowing using magnetoencephalography in 14 healthy subjects, and showed significant power decrease in the alpha and beta frequency ranges of event-related desynchronization within the sensorimotor area occurring at 5 min and faded 45 min after PES. Such alternation of cortical activity was associated with subtle, but significant improvements in swallowing performance during a water swallowing stress test [45•].

The effect of PES on swallowing was also tested by others in healthy subjects with different stimulation parameters. Takatsuji et al. [46] studied the effect of repetitive electrical pulse stimulation (0.2–1.2 mA, 1-ms pulse duration for 30 s) to the posterior wall of the oropharynx with different frequency (10–70 Hz) in ten healthy subjects. They found that the swallowing reflex was evoked successfully in all stimulation frequency, and the latency of the reflex became shorter as the stimulus frequency increased up to ≤ 30 Hz. Tsukano et al. [47] studied the effect of PES (30 Hz for 30 s, 80% of maximal tolerated intensity, 0.1 ms pulse duration) to different sites of pharynx (nasopharynx, oropharynx, and laryngopharynx) and to nasal cavity on repetitive voluntary swallowing (repetitive saliva-swallowing test) and on the swallowing reflex in 18 healthy subjects. Mean maximal tolerated intensity for each of stimulation sites ranged from 0.76 to 1.24 mA, and they demonstrated transient facilitation on both reflexive and voluntary swallowing by stimulation of nasopharynx, oropharynx, and laryngopharynx.

A series of studies by Hamdy and colleagues have clearly demonstrated that 5 Hz PES at 75% maximum tolerated intensity induces cortical plasticity with facilitative effects on swallowing. Indeed, considerable evidence showed that application of PES (at 5 Hz for 10 min over 3 days, mean stimulation amplitude 16.8 ± 6.6 mA) was associated with less radiological aspiration and clinical dysphagia in post-stroke dysphagic patients ([48•, 49] for review). However, it should be noted that stimulus intensity of PES was much higher in the studies conducted by Hamdy and colleagues [42–44] than that by others [46, 47]. Swallowing was induced reflexively in the latter studies, indicating that electrical stimulation conducted by Hamdy and colleagues activated more sensory nerves and/or receptors than that needed for swallow initiation (e.g., nociceptive receptor and/or fibers). It is also notable that PES at higher frequency (i.e., 10, 20, and 40 Hz) induced decrease in corticobulbar excitability [43]. Therefore, further studies will be needed to clarify the nature of sensory inputs generated by PES inducing cortical plasticity with facilitative and inhibitory effects on swallowing.

## Transcutaneous Electrical Stimulation

Another attempt to enhance swallowing initiation by peripheral electrical stimulation is application of transcutaneous electrical stimulation. Modalities of therapeutic transcutaneous electrical stimulation seem to be classified into two categories. One is neuromuscular electrical stimulation (NMES) that induces muscle contraction in the swallow-related muscles. This may include two expected effect; one is to strengthen the swallow-related muscles and the other to activate sensory afferent nerves, which usually induce transient, but in certain conditions, sustained effect within the central nervous system. The other is sensory afferent nerve stimulation, which focuses more on the latter effect. Although the muscle strengthening effect by NMES in healthy subjects is still uncertain, considerable evidence supports its effectiveness in weakened musculature, especially when NMES is applied together with voluntary activity [50]. There are a number of clinical studies to test the effect of NMES on swallowing performance or severity of dysphagia in patients (see [51, 52] for review). However, therapeutic effects of NMES seem to be inconsistent, and conflicting results have been reported, probably due to various electrodes and electrode placements, varying stimulation protocols, different aims, varying study protocols, and the various underlying pathological conditions [51].

In this review, we focus on the effects of sensory afferent nerve stimulation in healthy subjects and dysphasic patients, although the borderline between NMES and sensory afferent nerve stimulation are not clear regarding stimulation parameters. Gallas et al. [53] studied the effect of 1-h submental electrical stimulation (electrical trains: 5 s every minute, 80 Hz, under motor threshold) for 5 days in 11 dysphagic patients following a hemispheric (*n* = 7) or brainstem (*n* = 4) stroke. They showed that sensory afferent nerve stimulation (120% of the sensory threshold) induced an improvement in the subjective swallowing rating and swallowing reaction time and a decrease in aspiration and residue 1 week after stimulation. Also in nine healthy subjects, Cugy et al. [54•] showed that submental sensitive transcutaneous electrical stimulation (80 Hz for 20 min at 120% of the sensitive threshold: 11.8 ± 2.0 mA) reversed the inhibitory effects on MEPs and elongation of swallow reaction time induced by virtual lesion, and the effect was remained at 60 min after stimulation. They also showed that stimulation increased cortical excitability in the lesioned hemisphere. The findings suggest that sensory afferent nerve stimulation induces cortical plasticity and its therapeutic potential for post-stroke dysphagia.

Recently, Furuta et al. tested the effects of sensory afferent nerve stimulation to the neck on swallowing using another type of stimulation parameter the so-called interferential current (IFC) stimulation, consisting of carrier frequency and beat frequency in ten healthy subjects [55]. Carrier frequency is kHz-frequency alternating currents with low skin impedance allowing the current to reach deeper tissues [56] with less discomfort, and beat frequency is a low-frequency amplitude modulation responsible for therapeutic effects. They showed that surface IFC stimulation with 2 kHz carrier frequency and 50 Hz beat frequency at the sensory threshold significantly increased the number of swallows during 5 min stimulation period without discomfort. Although Furuta et al. reported that stimulation with pure kHz-frequency alternating currents (i.e., carrier frequency) did not affect swallowing, Jungheim et al. showed that 10-min medium-frequency (2.5 kHz) electrical stimulation to submandibular region at maximal tolerated intensity enhanced tongue base retraction during swallowing just after stimulation in 29 healthy subjects. So far, there is no evidence showing that transcutaneous electrical stimulation with medium frequency (more than 1 kHz) [51] or IFC induce “sustained” facilitative effect on swallowing. Transcutaneous electrical stimulation with such stimulation modality might be an alternative stimulation method in the treatment of dysphagia, but further study will be needed to verify its efficacy.

## Somatic and Chemical Stimulation

During natural feeding, sensory inputs initiating swallowing include somatic (e.g., touch and pressure), thermal and chemical (e.g., taste) ones, and usually arise from ingested food and liquid and activate sensory receptors. In this regard, somatic, thermal, and chemical stimulations can be considered more natural with less risk of adverse effects when stimulation intensity is kept within the physiological range compared to electrical stimulation.

Application of air pulse to the oropharynx is one of the recent approaches to elicit swallowing. Martin and colleagues found that oropharyngeal air-pulse stimulation increased resting swallowing rates (saliva swallowing) during 5 min stimulation period in four healthy young adults [57] and in 18 older adults [58] particularly when stimulation was applied bilaterally. Thereafter, they tested the effects of air-pulse stimulation (a total of six 10-s air-pulse trains at 2 Hz, air pressure 6–8 H_2_O) on swallowing in eight post-stroke dysphagic patients [59]. They then found that bilateral air-pulse stimulation induced increased resting swallowing rates during stimulation period in four patients with higher baseline (i.e., without stimulation) resting swallowing rates, but was not effective in remaining four patients with lower baseline swallowing rates. The findings provide an aspect of the limitation of such a “mild” sensory stimulation as an effective therapeutic intervention, depending on the severity of dysphasia, but suggest a potential usability of air-pulse stimulation as a tool for estimating the prognosis of patients.

Taste is one of the chemical sensory inputs that arise from ingested food and liquid and synapses predominantly in the nucleus NTS. Among taste qualities, use of sour bolus such as carbonated [60] and citric acid liquids (e.g., lemon juice) [61, 62] are the popular approaches for the treatment of dysphagia, and the studies reported therapeutic potential of using such stimuli in dysphagia rehabilitation. Mulheren et al. showed that a sour bolus (0.08 M citric acid) induces larger response in the cerebral cortex as well as increase in the frequency of swallowing to a greater degree than water and sweet stimuli in 15 healthy subjects [63]. Recently, Elshukri et al. tested the effects of 5-ml liquid bolus swallows of carbonated, citric acid, and still water on volitional swallowing performance and corticobulbar excitability evaluated by pharyngeal MEPs in healthy adults [64•]. Results showed improvement in swallowing performance (number of correctly timed swallows within a predetermined time window) mainly with carbonated liquid. Also, stimuli with carbonated and citric acid liquids induced sustained increase in corticobulbar excitability up to 1 h, which was more prominent for carbonated liquid. The finding indicates that sour stimuli with carbonated and citric acid liquids induce cortical plasticity with facilitative effects on swallowing. It should be noted however, several evidence suggests that the facilitative of swallowing is due to activation of nociceptive pathways ([65, 66] see [67] for review). For this, further studies will be needed to clarify the nature of sensory inputs generated by effective sour stimuli.

## Inhibitory Effects on Swallowing Induced by Peripheral Stimulation

Although it is generally considered that stimulation of greater number of receptive fields and their sensory neurons induces a stronger swallowing response, there are several lines of evidence that some sensory inputs inhibiting swallowing initiation. For example, electrical stimulation of the lingual branch of the glossopharyngeal nerve innervating posterior tongue mucosa inhibits the swallowing reflex in experimental animals [68]. Also in healthy subjects, application of small amount of salty (sodium chloride: NaCl) or bitter (qunine-HCl) liquids to the oropharynx inhibit swallowing initiation [67].

## Conclusions

We have introduced recent approaches enhancing swallowing initiation particularly with neuroplastic changes: central interventions such as non-invasive brain stimulation and peripheral interventions such as peripheral electrical stimulation and natural stimulation. For the central interventions, studies support the view that 5-Hz rTMS to the cortical motor area is an effective approach for the patients with unilateral brain damage by enhancing a motor recovery as long as the targeted area is properly mapped, although the mapping within the damaged hemisphere may be difficult. tDCS also has a therapeutic potential similar to rTMS, but careful clinical observation will be needed to verify possible risk of adverse effects when stimulation spread to surrounding the targeted area. For the peripheral interventions, 5-Hz PES at 75% maximum tolerated intensity may be an effective intervention enhancing the corticobulbar excitability. Other interventions need further studies to verify their efficacy, for example, duration of the effect and a limit of effectiveness depending on pathological conditions. In addition, studies clarifying the nature of peripheral inputs inducing sustained facilitative effects on swallowing will be expected.

We also introduced recent findings showing central and peripheral inputs inhibiting swallow initiation. It may be important to avoid such inputs during meal assistance. Recently, studies to test the effects of combined intervention on swallowing have started. It seems that combination of effective interventions may not necessarily to induce a better effect (e.g., [69]). Further studies are also expected.
